# Genetic Deletion of P2Y_2_ Receptor Offers Long-Term (5 Months) Protection Against Lithium-Induced Polyuria, Natriuresis, Kaliuresis, and Collecting Duct Remodeling and Cell Proliferation

**DOI:** 10.3389/fphys.2018.01765

**Published:** 2018-12-17

**Authors:** Yue Zhang, Anne Riquier-Brison, Tao Liu, Yufeng Huang, Noel G. Carlson, János Peti-Peterdi, Bellamkonda K. Kishore

**Affiliations:** ^1^Nephrology Research, Department of Veterans Affairs Salt Lake City Health Care System, Salt Lake City, UT, United States; ^2^Department of Internal Medicine, University of Utah Health, Salt Lake City, UT, United States; ^3^Jiangsu Key Laboratory of Pediatrics, Nanjing Medical University, Nanjing, China; ^4^Zilkha Neurogenetic Institute, University of Southern California, Los Angeles, CA, United States; ^5^Department of Physiology and Neuroscience, University of Southern California, Los Angeles, CA, United States; ^6^Geriatric Research, Education and Clinical Center, Department of Veterans Affairs Salt Lake City Health Care System, Salt Lake City, UT, United States; ^7^Department of Neurobiology and Anatomy, University of Utah Health, Salt Lake City, UT, United States; ^8^Center on Aging, University of Utah Health, Salt Lake City, UT, United States; ^9^Department of Nutrition and Integrative Physiology, University of Utah Health, Salt Lake City, UT, United States

**Keywords:** diabetes insipidus, polyuria, natriuresis, kaliuresis, collecting duct remodeling, cell proliferation, purinergic receptors

## Abstract

Chronic lithium administration for the treatment of bipolar disorder leads to nephrogenic diabetes insipidus (NDI), characterized by polyuria, natriuresis, kaliuresis, and collecting duct remodeling and cell proliferation among other features. Previously, using a 2-week lithium-induced NDI model, we reported that P2Y_2_ receptor (R) knockout mice are significantly resistant to polyuria, natriuresis, kaliuresis, and decrease in AQP2 protein abundance in the kidney relative to wild type mice. Here we show this protection is long-lasting, and is also associated with significant amelioration of lithium-induced collecting duct remodeling and cell proliferation. Age-matched wild type and knockout mice were fed regular (*n* = 5/genotype) or lithium-added (40 mmol/kg chow; *n* = 10/genotype) diet for 5 months and euthanized. Water intake, urine output and osmolality were monitored once in every month. Salt blocks were provided to mice on lithium-diet to prevent sodium loss. At the end of 5 months mice were euthanized and serum and kidney samples were analyzed. There was a steady increase in lithium-induced polyuria, natriuresis and kaliuresis in wild type mice over the 5-month period. Increases in these urinary parameters were very low in lithium-fed knockout mice, resulting in significantly widening differences between the wild type and knockout mice. Terminal AQP2 and NKCC2 protein abundances in the kidney were significantly higher in lithium-fed knockout vs. wild type mice. There were no significant differences in terminal serum lithium or sodium levels between the wild type and knockout mice. Confocal immunofluorescence microscopy revealed that lithium-induced marked remodeling of collecting duct with significantly increased proportion of [H^+^]-ATPase-positive intercalated cells and decreased proportion of AQP2-positive principal cells in the wild type, but not in knockout mice. Lithium-induced collecting duct cell proliferation (indicated by Ki67 labeling), was significantly lower in knockout vs. wild type mice. This is the first piece of evidence that purinergic signaling is potentially involved in lithium-induced collecting duct remodeling and cell proliferation. Our results demonstrate that genetic deletion of P2Y_2_-R protects against the key structural and functional alterations in Li-induced NDI, and underscore the potential utility of targeting this receptor for the treatment of NDI in bipolar patients on chronic lithium therapy.

## Introduction

About 4% of the 21.8 million United States Veterans suffer from bipolar disorder, a sequel of post-traumatic stress disorder (PTSD). Currently about 30% of bipolar Veterans receive lithium therapy, where it has distinct advantages over the non-lithium drugs. About 25–50% of bipolar patients attempt suicide at least once (Jamison, 2000), and lithium is very effective in countering suicidal tendencies in bipolar patients (Jamison, 2000; Baldenssarini et al., 2006; Nivoli et al., 2010; Chiu et al., 2013). Other drugs have not proven to be more efficacious than lithium in this respect (DeLa Crux et al., 2003; Ahearn et al., 2013). Furthermore, in recent years lithium has emerged as a robust neuroprotective agent for the treatment of acute brain injury and chronic neurodegenerative diseases (Rowe and Chuang, 2004; Wada et al., 2005; Quiroz et al., 2010; Florenza et al., 2012; Chiu et al., 2013). Thus, beyond its current use in bipolar disorder, the neuroprotective ability of lithium implies it could be used to treat or prevent brain damage following acute injury, such as ischemic stroke or chronic neurodegenerative diseases, such as Alzheimer’s, Parkinson’s, or Huntington’s disease.

Despite its superior therapeutic potential, chronic administration of lithium for bipolar disorder is often limited by its adverse effects on the kidney (reviewed in Grünfeld and Rossier, 2009; Kishore and Ecelbarger, 2013), resulting in the development of nephrogenic diabetes insipidus (NDI). Patients with lithium-induced NDI present with polyuria, natriuresis and kaliuresis, reduced ability to concentrate urine, and are unresponsive to the administration of arginine vasopressin (AVP). Another salient feature of lithium-induced NDI as demonstrated in rodent models, is remodeling of the collecting duct, whereby the proportion of AQP2-positive principal cells (PC) decrease and the proportion of [H^+^]-ATPase-positive intercalated cells (IC) increase, associated with proliferation of primarily principal cells (Christensen et al., 2004, 2006). Clinically, in addition to significant social inconvenience, NDI causes considerable morbidity and even mortality. Elderly patients with NDI have an elevated risk of dehydration, hypernatremia, alterations in consciousness, and hemodynamic instability from hypovolemia (Luckey and Parsa, 1995; Rej et al., 2012).

Currently used modalities for the treatment of NDI, such as the combined use of a thiazide and amiloride or non-steroidal anti-inflammatory drugs (NSAID; indomethacin or selective COX inhibitors) are encountered with varying degrees of success as well as side effects. Thiazides can cause lithium intoxication, whereas the use of amiloride enhances lithium-induced natriuresis (Finley et al., 1995; Bedford et al., 2008; Kortenoeven et al., 2009). Indomethacin is not tolerated well by many patients (Murray and Brater, 1993; Phelan et al., 2003), and chronic inhibition of cyclooxygenases (COX) is not safe (Conaghan, 2012). Hence, there is a need for better and safer therapeutic methods to treat lithium-induced NDI.

In the above context, we used a rat model to discover that extracellular ATP/UTP-activated P2Y_2_ receptor is involved in the development of lithium-induced NDI (Zhang et al., 2009). Since no FDA-approved drugs that can selectively block P2Y_2_ receptor are available, we used a genetic deletion model and showed that blunting of this receptor confers significant protection against lithium-induced polyuria, natriuresis and kaliuresis, and significantly preserves AQP2 protein abundance, without causing lithium-intoxication (Zhang et al., 2012, 2013). In these studies, we used a 2-week lithium-fed model. However, treatment of bipolar disorder by the administration of lithium is a chronic process, lasting for years or decades. Thus, the aim of the current study is to determine whether genetic deletion of P2Y_2_ receptor offers long-term protection against lithium-induced NDI. Specifically, using a model of lithium administration for 5 months we evaluated the beneficial effects of genetic deletion of P2Y_2_ receptor on lithium-induced polyuria, natriuresis, kaliuresis, AQP2 protein abundance in the kidney, as well as remodeling of collecting duct and proliferation of medullary collecting duct cells.

## Materials and Methods

### Experimental Animals and Protocol

The animal procedures described here were approved by the Institutional Animal Care and Use Committee of the Veterans Affairs Salt Lake City Health Care System, where the animals were housed. Breeders of P2Y_2_ receptor knockout mice (B6D2 genetic background) were obtained from Dr. Beverly Koller (University of North Carolina at Chapel Hill, Chapel Hill, NC, United States) (Cressman et al., 1999; Homolya et al., 1999). Breeding and genotypic evaluation of the mice were described previously (Zhang et al., 2008). Groups of age-matched adult wild type (WT) and knockout (KO) mice were fed either regular rodent chow (*n* = 5 mice/genotype) or lithium-added chow (40 mmol LiCl/kg chow; MP Biomedicals, Solon, OH, United States; *n* = 10 mice/genotype). All mice had free access to food and drinking water during the entire experimental period. Salt blocks were provided to the mice feeding lithium diet to prevent sodium depletion. The feeding lasted for 5 months. Once in every month the mice were kept in plastic metabolic cages (1 mouse/cage) for 2 consecutive days and 24-h water intake and urine outputs were determined. At the end of the experimental period all mice were humanely euthanized and blood and kidney tissues were collected and processed for analysis.

### Blood and Urine Analysis

Urine volumes were recorded and osmolalities of clear urine and serum samples were determined by vapor pressure method (Wescor, Logan, UT, United States). Concentrations of sodium, potassium and lithium in urine and/or serum samples were measured on Easy*Lyte* (Medica, Bedford, MA, United States). Serum urea concentration was determined by an enzymatic method using a commercial kit (BioVision, Milpitas, CA, United States).

### Analysis of Kidney Tissue Samples for Protein Abundances

This was performed by semi-quantitative immunoblotting (Western blotting), essentially as described previously (Zhang et al., 2012, 2013, 2015a,b). Briefly, cortical and whole medullary tissues were homogenized in a buffer containing protease inhibitors. After determination of protein concentrations, the homogenates were solubilized in Laemmli buffer. Proteins in the homogenates were size fractionated by electrophoresis on 12% polyacrylamide gels (Life Technologies, Grand Island, NY, United States), and then electro-transferred on to nitrocellulose membranes (Life Technologies). Rabbit polyclonal antibodies to aquaporin-2 (AQP2), and bumetanide-sensitive Na-K cotransporter (NKCC2) were used in Western blot analysis to determine the protein abundances of AQP2 and NKCC2. Generation and characterization of these two antibodies has been described previously (Kishore et al., 2000; Kishore and Ecelbarger, 2013; Zhang et al., 2013). Protein abundance of β-actin in the samples was used as internal control for loading. β-actin antibody was purchased from BioLegend (San Diego, CA, United States). Peroxidase conjugated secondary antibody against rabbit IgG (Dako North America, Inc., Carpinteria, CA, United States) was used in conjunction with chemiluminescence reagent (SuperSignal, Pierce Endogen, Rockford, IL, United States). Sites of antigen-antibody reaction were captured on X-ray films. Images were digitized, and band densities were determined by Un-Scan-It software (Silk Scientific, Orem, UT, United States). AQP2 band densities were normalized to the corresponding band densities of β-actin, and expressed as percent of the mean values in the control diet fed WT mice.

### Histopathological Examination of the Kidney

Freshly obtained kidneys from WT and KO mice were cut transversely into two halves, and one half was fixed in 10% buffered-formalin for a few days, dehydrated and then embedded in paraffin. The paraffin-embedded kidneys were cut into 5 μm thick sections, deparaffinized, rehydrated and stained with hematoxylin-eosin or PAS (periodic acid-Schiff reagent) or with Masson’s trichrome stain. Stained sections were examined under a Reichert light microscope, and digital pictures were taken with a Nikon 995 Coolpix camera.

### Immunofluorescence Microscopy for Matrix Proteins

Deparaffinized and rehydrated kidney sections obtained as above were treated with Dako target retrieval solution (Dako North America) for antigen retrieval. Then the sections were processed for immunofluorescence detection of α-smooth muscle actin (α-SMA) and fibronectin (FN) as described previously (Huang et al., 2008; Tang et al., 2017). Sections were incubated with either monoclonal anti-α -SMA IgG2a (Cat # No. A2547; 1:500 dilution; Sigma Chemical Co., St. Louis, MO, United States), or rabbit anti-human fibronectin (Cat No. F3648; 1:300 dilution; Sigma Chemical Co.) as the primary antibodies at 4°C overnight. Red^TM^-X-conjugated donkey anti-mouse IgG and FITC-conjugated donkey anti-rabbit IgG (Jackson ImmunoResearch Laboratories Inc., West Grove, PA, United States; 1:200 dilution) were applied as the secondary antibodies at room temperature for 2 h. Control slides treated with antibody diluent instead of primary antibody showed no staining.

### Confocal Immunofluorescence Analysis of Collecting Duct Remodeling and Cell Proliferation

Deparaffinized and rehydrated kidney sections were processed for immunofluorescence as described previously (Zhang et al., 2015b). Briefly, to retrieve antigens, sections were immersed in phosphate buffered saline (PBS) and heated in a microwave oven (2 min × 10 min) at medium power, and then allowed to cool for 40 min. Sections were then permeabilized with 0.1% Triton X-100 in PBS for 10 min, followed by blocking with goat serum (1:200 dilution, Jackson ImmunoResearch Laboratories, Inc., West Grove, PA, United States). Sections were then probed with AQP2 goat polyclonal antibody (1:100 dilution; sc-9882 from Santa Cruz, CA, United States) followed by donkey anti-goat secondary Alexa fluor 488-conjugated antibody (1:500 dilution). Some sections were double labeled with either polyclonal antibody to [H^+^]-ATPase, generous gift from Dr. Mark Knepper (Christensen et al., 2004), or Ki67 antibody (1:200 dilution; 14-5698 from eBioscience, San Diego, CA, United States), followed by donkey anti-rabbit secondary Alexa fluor 594-conjugated antibody (1:500 dilution).

### Statistical Analysis

All quantitative data were expressed as mean ± standard error of the mean (SE). Initial statistical analysis was done by one-way analysis of variance (ANOVA) followed by the assessment of significance by the Tukey-Kramer Multiple Comparison Test or Bonferroni Comparison Test for groups that passed normality tests. GraphPad InStat^®^ software (GraphPad Software, La Jolla, CA, United States) was used for these statistical analyses. For the simplicity of presentation, statistical significances between the groups obtained by this method are used in the figures and figure legends. Further analysis was performed to find differences due to main factors, the phenotype and treatment with lithium and the interaction of these two factors by two-way ANOVA, using SigmaPlot^®^ software (Systat Software, Inc., CA, United States). Differences between individual pairs of means were determined by a Pairwise Multiple Comparison Procedure (Holm-Sidak method) after a significant (*P* < 0.05) one-way ANOVA. For each parameter analyzed, four different *P*-values were generated corresponding to the four factors, namely: (i) effect of lithium diet in WT mice; (ii) effect of lithium in KO mice; (iii) interaction of phenotype with control diet; and (iv) interaction of phenotype with lithium diet. These *P*-values are shown in Table 1. When comparing the means of two different groups directly, an unpaired *t*-test was used. *P*-values less than 0.05 were considered significant.

**Table 1:** *P*-values for the two-way analysis of variance of the data.

Parameter	Effect of lithium diet in		Interaction of phenotype with	
	Wild type mice	Knockout mice	Control diet	Lithium diet
Water intake	*P* < 0.001	*P* = 0.192	*P* = 0.499	*P* = 0.036
Urine output	*P* < 0.001	*P* = 0.325	*P* = 0.293	*P* < 0.001
Urine osmolality	*P* < 0.001	*P* = 0.310	*P* = 0.276	*P* < 0.001
Urine sodium	*P* < 0.001	*P* < 0.001	*P* = 0.976	*P* < 0.001
Urine potassium	*P* < 0.001	*P* = 0.440	*P* = 0.669	*P* < 0.001
AQP2 in medulla	*P* < 0.001	*P* = 0.017	*P* = 0.487	*P* < 0.001
AQP2 in cortex	*P* < 0.001	*P* < 0.001	*P* < 0.001	*P* < 0.003
NKCC2 in medulla	*P* < 0.001	*P* = 0.166	*P* = 0.324	*P* = 0.103
NKCC2 in cortex	*P* = 0.203	*P* = 0.001	*P* = 0.012	*P* = 0.976
PC in medulla	*P* < 0.001	*P* = 0.278	*P* = 0.447	*P* = 0.001
PC in cortex	*P* = 0.025	*P* = 0.459	*P* = 0.888	*P* = 0.006
IC in medulla	*P* < 0.001	*P* = 0.316	*P* = 0.526	*P* < 0.001
IC in cortex	*P* < 0.010	*P* = 0.404	*P* = 0.880	*P* = 0.002

*PC, principal cells; IC, intercalated cells.*

## Results

### Effect of Long-Term Lithium Administration on Water Metabolism

**FIGURE 1:**
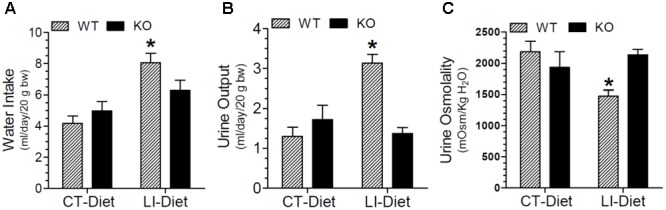
Terminal water intake **(A)**, urine output **(B)**, and urine osmolality **(C)** in WT and P2Y_2_ KO mice fed control or lithium-added diet for 5 months. Values shown are mean ± se (CT, *n* = 5 mice/genotype; LI, *n* = 10 mice/genotype) of two consecutive days just prior to euthanasia. ^∗^Significantly different from the CT-Diet fed WT mice and LI-Diet fed KO mice groups by one-way ANOVA. For the results of two-way ANOVA refer to Table 1.

**FIGURE 2:**
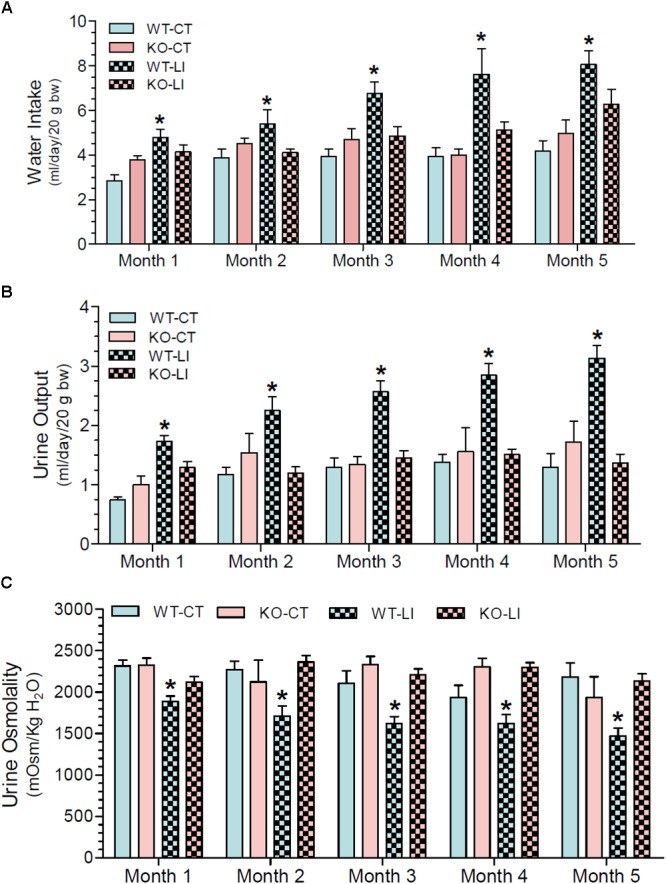
Monthly record of water intake **(A)**, urine output **(B)**, and urine osmolality **(C)** in WT and P2Y_2_ KO mice fed control or lithium-added diet for 5 months. Values shown are mean ± se (CT, *n* = 5 mice/genotype; LI, *n* = 10 mice/genotype) of two consecutive. One-way ANOVA: Water intake: ^∗^significantly different from the corresponding WT-CT at all time points, and from the corresponding KO-LI at all time points except month 1. Urine output: ^∗^significantly different from the corresponding WT-CT and KO-LI at all time points. Urine osmolality: ^∗^significantly different from the corresponding WT-CT and KO-LI at all time points.

The effect of long-term lithium administration on water metabolism was assessed by the determination of 24-h water intake, urine output and osmolality once every month for 5 months. Together these parameters indicate the severity of lithium-induced NDI due to impairment of urinary concentrating mechanism. Figure 1 shows the terminal water intake, urine output and osmolality determined after 5 months of treatment just prior to euthanasia. Two-way ANOVA revealed that there is significant interaction between the phenotype and lithium diet for all three parameters (Table 1). Figure 2 shows the same parameters recorded on each month. As shown in Figure 1, after 5 months of lithium feeding, there were significant increases in water intake and urine output, associated with significant decrease in urine osmolality in the WT mice, but not in the P2Y_2_ KO mice. This resulted in significant differences in lithium-fed WT vs. KO mice in all three parameters. Thus, even after 5 months, the P2Y_2_ KO mice were significantly resistant to lithium-induced polyuria. Furthermore, when the changes in these parameters were considered over the time (Figure 2), it is obvious that the magnitude of increases in water intake and urine output as well as the decrease in urine osmolality were progressive. On the other hand, these parameters remained steady or showed minimal changes in lithium-fed KO mice, resulting in significant differences between the genotypes over the experimental period (Figure 2).

### Terminal AQP2 Protein in the Kidney

**FIGURE 3:**
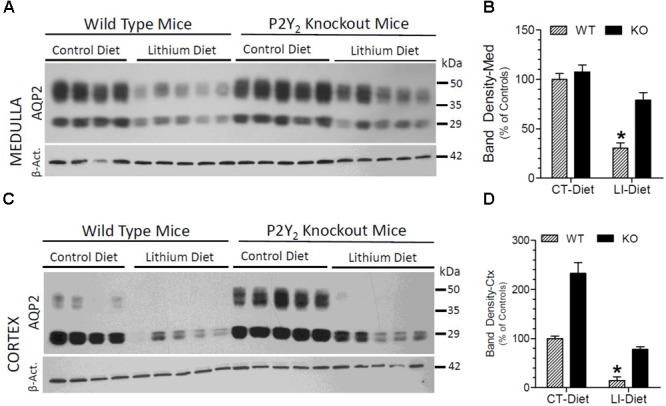
Terminal (5 months) protein abundances of AQP2 in the kidney of mice fed control or lithium-added diet determined by Western blotting. **(A)** Immunoblot profile of AQP2 and β-actin protein bands in the renal medulla of different groups of mice. **(B)** Mean densitometric values of normalized AQP2 and β-actin protein bands in the medulla expressed as percent of mean values in the control diet fed WT mice. **(C)** Immunoblot profile of AQP2 and β-actin protein bands in the renal cortex of different groups of mice. **(D)** Mean densitometric values of normalized AQP2 and β-actin protein bands in the cortex expressed as percent of mean values in the control diet fed WT mice. ^∗^Significantly different from the CT-Diet fed WT and LI-Diet fed KO mice by one-way ANOVA. For results of two-way ANOVA, refer to Table 1. WT-CT, wild type mice on control diet; WT-LI, wild type mice fed lithium-added diet; KO-CT, knockout mice on control diet; KO-LI, knockout mice on lithium-added diet.

Semi-quantitative immunoblotting was used to determine AQP2 protein abundances in the renal cortex and medulla of control or lithium diet fed WT and P2Y_2_ KO mice at the end of 5 months treatment. The protein abundance of β-actin was used as internal control for each sample. As expected, lithium-feeding caused marked decrease in AQP2 protein band densities in the renal medulla and cortex of WT mice as compared to the control diet-fed WT mice (Figure 3). However, the mean values in the KO mice were 2.7- and 5-fold higher, respectively, as compared to the lithium-fed WT mice (Figures 3B,D). Furthermore, the protein abundances of AQP2 in control diet-fed KO mice were about 2.4-fold higher as compared to the corresponding value in control diet-fed WT mice (Figure 3D). Thus, despite 5 months of lithium-administration AQP2 protein abundances in the kidneys of P2Y_2_ KO mice is well preserved. Accordingly, two-way ANOVA revealed significant interaction between the phenotype and lithium diet with respect to AQP2 protein abundance in the kidney (Table 1).

### AQP2 Protein Cellular Disposition in the Renal Medulla

**FIGURE 4:**
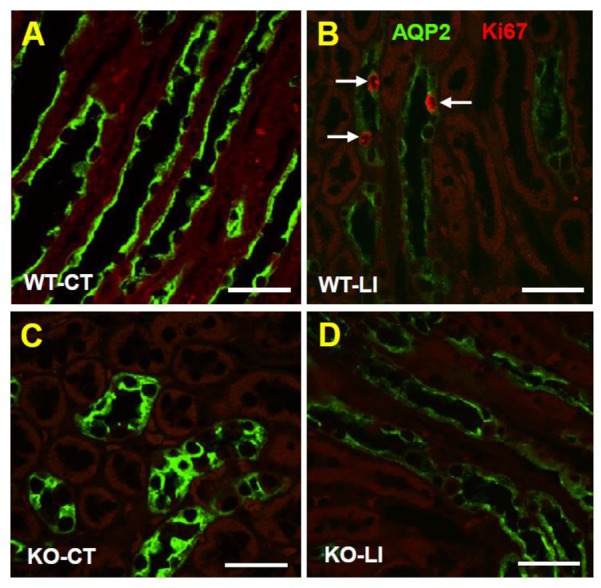
Immunofluorescence imaging of cellular expression and disposition of AQP2 protein in the renal medulla of WT and KO mice fed control or lithium-added diets for 5 months. Representative images from the renal medulla are shown. Sections were co-stained for AQP2 (green) and the Ki67 (red), a nuclear cell marker for proliferation. Note the diminished AQP2 immunofluorescence in response to lithium-treatment in WT but not in KO mice, and the presence of Ki67+ cells in lithium-treated WT mice. WT-CT **(A)**, wild type mice on control diet; WT-LI **(B)**, wild type mice fed lithium-added diet; KO-C **(C)**, knockout mice on control diet; KO-LI **(D)**, knockout mice on lithium-added diet. Bar is 20 μm.

Confocal immunofluorescence microscopy revealed that administration of lithium to WT mice for 5 months resulted in marked decrease in AQP2 immunofluorescence in the collecting ducts of renal medulla, with the accumulation of most of the remaining protein at the apical domain (Figure 4B). In contrast, administration of lithium to the P2Y_2_ KO mice showed preservation of more AQP2 protein, which was distributed within the collecting duct cells, although the total amount of AQP2 protein in lithium-fed KO mice appears to be much less (Figure 4D) as compared to the renal medulla of control diet-fed KO mice (Figure 4C).

### Effect of Long-Term Lithium Administration on Natriuresis and Kaliuresis

**FIGURE 5:**
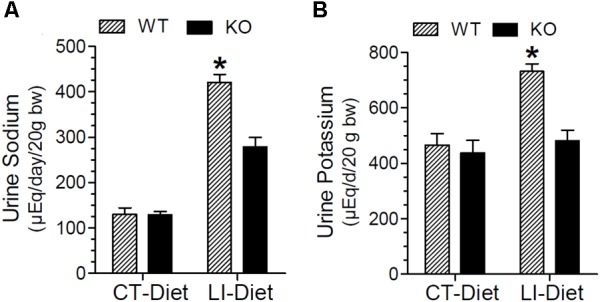
Terminal urine sodium **(A)** and potassium **(B)** in WT and P2Y2 KO mice fed regular or lithium-added diets for 5 months. Values shown are mean ± se (CT; *n* = 5 mice/genotype; LI; *n* = 10 mice/genotype). Statistical significance between the groups is shown over the bars. ^∗^Significantly different from the CT-Diet fed WT mice and LI-Diet fed KO mice groups by one-way ANOVA. For the results of two-way ANOVA refer to Table 1.

**FIGURE 6:**
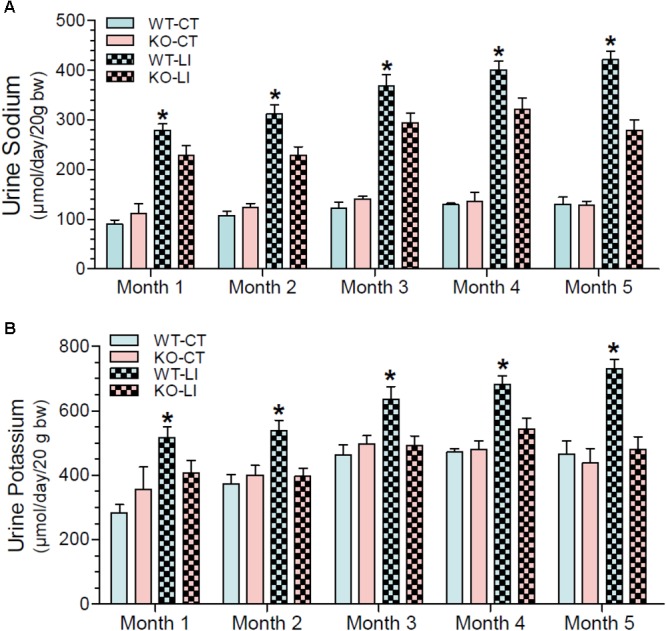
Monthly record of water intake urine sodium **(A)** and potassium **(B)** in WT and P2Y_2_ KO mice fed control or lithium-added diet for 5 months. Values shown are mean ± se of 24-h samples (CT, *n* = 5 mice/genotype; LI, *n* = 10 mice/genotype). ^∗^Significantly different from the corresponding WT-CT and KO-LI at all time points by one-way ANOVA.

In addition to polyuria, lithium administration causes natriuresis and kaliuresis. Previously, using a 2-week model, we showed that P2Y_2_ KO mice are significantly resistant to lithium-induced natriuresis and kaliuresis (Zhang et al., 2012). Hence, we examined whether the P2Y_2_ KO mice are resistant to lithium-induced natriuresis and kaliuresis in the long-term model also. Figure 5 shows the urinary excretion of sodium or potassium after lithium-treatment for 5 months. Figure 6 shows the same parameters for each month of the experimental period. As shown in the Figure 5, lithium treatment for 5 months caused significant 3.2- and 1.6-fold increase in urinary excretion of sodium and potassium, respectively, in the WT mice. But the corresponding increases in lithium-fed KO mice were minimal (1.9-fold for sodium) or absent (potassium) (Figure 5). Accordingly, two-way ANOVA revealed significant interaction between the phenotype and lithium diet for both natriuresis and kaliuresis (Table 1). Examination of the urinary excretion of sodium and potassium over the time showed that lithium-induced increases in WT mice progressive in WT mice, but not in the P2Y_2_ KO mice (Figure 6).

### Terminal NKCC2 Protein Abundance in the Kidney

**FIGURE 7:**
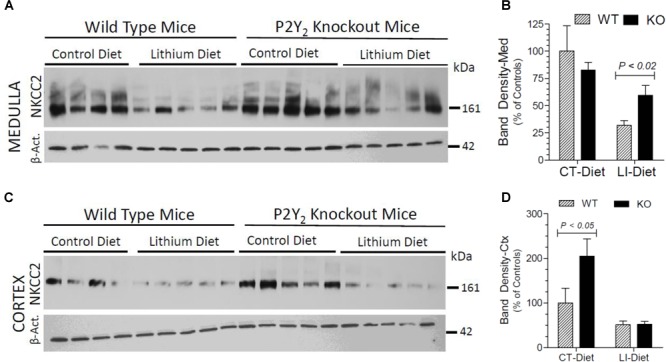
Terminal (5 months) protein abundances of NKCC2 in the kidney of mice fed control or lithium-added diet determined by Western blotting. **(A)** Immunoblot profile of NKKC2 and β-actin protein bands in the renal medulla of different groups of mice. **(B)** Mean densitometric values of normalized NKCC2 and β-actin protein bands in the medulla expressed as percent of mean values in the control diet fed WT mice. **(C)** Immunoblot profile of NKKC2 and β-actin protein bands in the renal cortex of different groups of mice. **(D)** Mean densitometric values of normalized NKCC2 and β-actin protein bands in the cortex expressed as percent of mean values in the control diet fed WT mice. Statistical significance between the groups by one-way ANOVA is shown over the bars. For the results of two-way ANOVA refer to Table 1.

In order to understand the contribution of bumetanide-sensitive Na-K-Cl co-transporter-2 (NKCC2) of the thick ascending limb to the observed protection against lithium-induced natriuresis in P2Y_2_ KO mice, we determined its terminal protein abundance by Western blot analysis. As shown in Figure 7, lithium-treatment caused marked decrease in the mean protein abundance of NKCC2 in the renal medulla of WT mice. But the corresponding decrease in lithium-fed KO mice was modest, with the result that the KO mice still had 1.9-fold higher protein abundance as compared to the WT mice (Figure 7B). In contrast, in the cortex lithium administration caused significant decreases in NKCC2 protein abundance in both WT and KO mice with no difference in their mean values (Figure 7D). Interestingly, similar to AQP2 protein abundance, the mean NKCC2 protein abundance in control diet-fed P2Y_2_ KO mice was 2-fold higher as compared to the corresponding value in WT mice (Figure 7D). Two-way ANOVA revealed significant interaction between the phenotype and control diet for NKCC2 protein abundance in renal cortex (Table 1).

### Effect of Long-Term Lithium Administration on Terminal Blood Parameters

**FIGURE 8:**
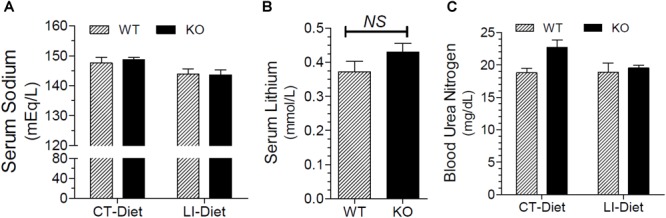
Terminal blood parameters in mice fed control of lithium-added diets for 5 months. **(A)** Serum sodium levels in WT and KO mice fed control or lithium-added diets. **(B)** Serum lithium levels in lithium-fed WT and KO mice. **(C)** Blood urea nitrogen (BUN) levels in WT or KO mice fed control of lithium-added diets.

Figure 8A shows that terminal serum sodium levels in lithium-fed WT and KO mice were not different, and were within the normal limits. Figure 8B shows that terminal serum lithium levels were not significantly different between the two genotypes. Mean blood urea nitrogen (BUN) levels in lithium of control diet-fed WT and KO mice were comparable and within the normal limits, although the mean value in control diet fed KO was numerically higher (Figure 8C).

### Effect of Long-Term Lithium Administration on Histopathology of the Kidney and on Matrix Proteins

**FIGURE 9:**
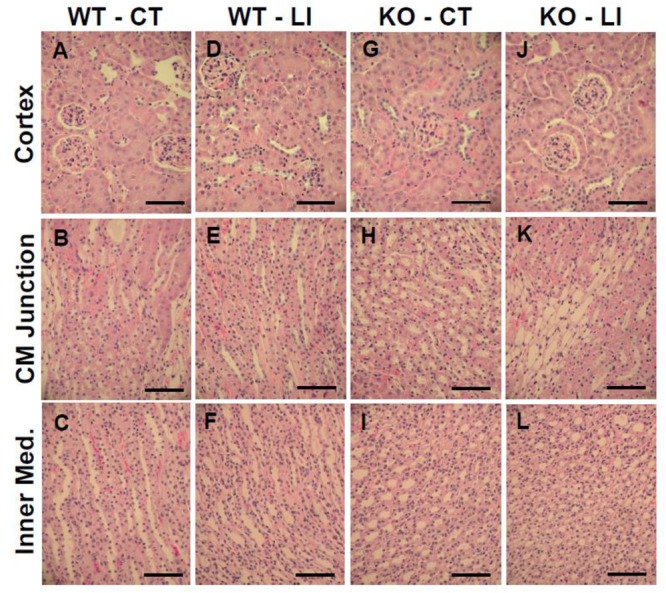
Representative profiles of morphological appearance of kidneys of WT and P2Y_2_ KO mice fed control (CT) or lithium-added (LI) diets for 5 months. Formalin-fixed and paraffin-embedded kidney samples were sectioned and stained with hematoxylin-eosin. **(A–C)** WT control mice; **(D–F)** WT lithium mice; **(G–I)** KO control mice; and **(J–L)** KO lithium mice. CM Junction – cortico medullary junction. Bar represents 50 μm.

**FIGURE 10:**
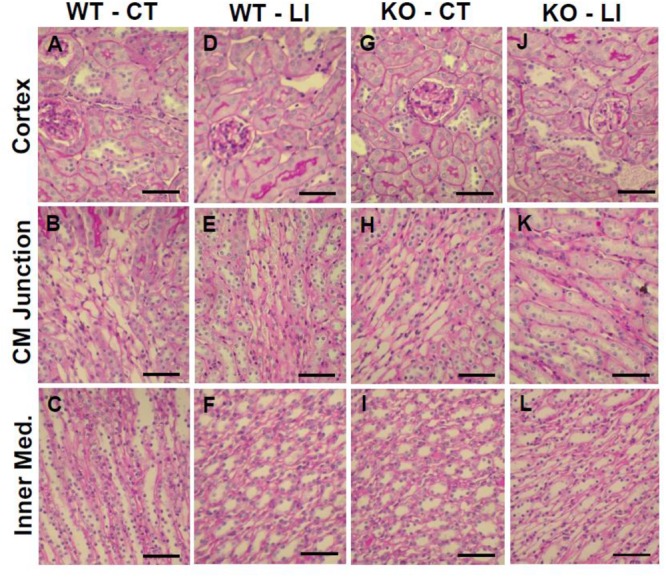
Representative profiles of morphological appearance of kidneys of WT and P2Y_2_ KO mice fed control (CT) or lithium-added (LI) diets for 5 months. Formalin-fixed and paraffin-embedded kidney samples were sectioned and stained with PAS (periodic acid Schiff reagent). **(A–C)** WT control mice; **(D–F)** WT lithium mice; **(G–I)** KO control mice; and **(J–L)** KO lithium mice. CM Junction – cortico-medullary junction. Bar represents 50 μm.

**FIGURE 11:**
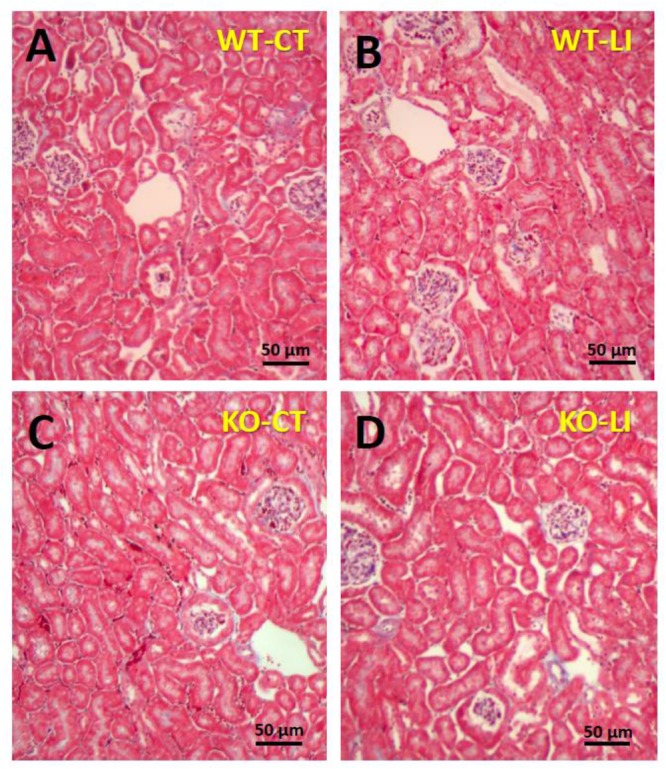
Representative profiles of Masson’s trichrome stained kidney sections of WT and P2Y_2_ KO mice fed control (CT) or lithium-added (LI) diets for 5 months. Formalin-fixed and paraffin-embedded kidney samples were sectioned and stained with Masson’s trichrome reagent. **(A)** WT control mouse; **(B)** WT lithium mouse; **(C)** KO control mouse; and **(D)** KO lithium mouse.

The panels in Figures 9–11 show representative light microscopic profiles of different regions of the kidneys, such as cortical, cortico-medullary and inner medulla, from the WT and KO mice stained with hematoxylin-eosin (H&E) or PAS or Masson’s trichrome. H&E stained sections did not reveal any discernible differences in the morphology of the kidneys from WT vs. KO mice (Figure 9). Similarly, no abnormalities in the integrity of cell membranes could be detected between the WT and KO mice kidneys in PAS-stained sections (Figure 10). Finally, no detectable signs of fibrosis could be seen in the sections stained with Masson’s trichrome stain (Figure 11).

We also probed for potential lithium-induced alterations in the matrix proteins in the kidney. Immunofluorescence microscopy for α-smooth muscle actin and fibronectin did not reveal detectable alterations in extracellular matrix in the kidney after lithium-feeding (data not shown). Obviously, longer duration of treatment using higher doses of lithium (60 mmol/Kg chow) is needed to induce such structural changes in the extracellular matrix (Walker et al., 2013).

### Effect of Long-Term Lithium Administration on Collecting Duct Remodeling

**FIGURE 12:**
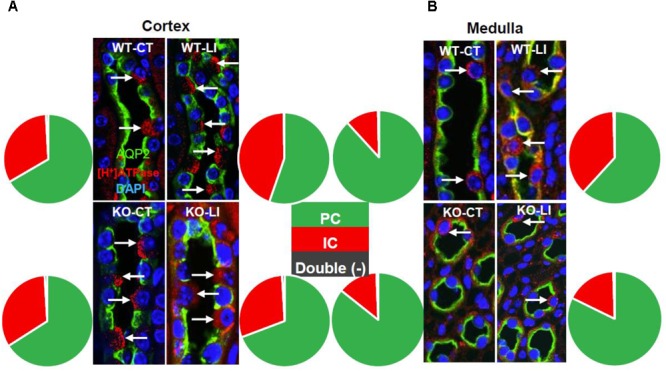
Representative images and summary of the confocal immunofluorescence analysis of chronic Li treatment-induced changes in cortical **(A)** and medullary collecting duct **(B)** cell composition in wild type (WT) or P2Y_2_ knockout (KO) control (CT) or lithium-fed (LI) mice. AQP2 (green) and [H^+^]-ATPase (red) double-immunolabeling was used to identify the principal (PC) and intercalated (IC) cells of the collecting duct, respectively. Nuclei are stained with DAPI. Note the significantly increased number of ICs in lithium-treated wild type mice in both cortex and medulla, but only a blunted response in the knockout mice, as illustrated by pie charts. A detectable, but negligible fraction of cells (0.2–0.9%) were stained double negative (black slice in pie charts). For mean ± SE of percent changes and statistical significance by one-way ANOVA refer to Table 1. For the results of the two-way ANOVA refer to Table 2.

Another hallmark of lithium effect on the kidney is collecting duct remodeling, whereby the proportion of AQP2-positive principal cells (PC) are reduced with a corresponding increase in [H^+^]-ATPase-positive intercalated cells (IC). Hence, using confocal immunofluorescence microscopy we enumerated the cells that stained for AQP2 (green) or [H^+^]-ATPase (red) or none in the cortical and medullary collecting ducts of WT and KO mice fed either control or lithium-added diets. Cell nuclei were stained with DAPI (blue). Figure 12 shows representative images of confocal microscopy with double labeling, and pie charts for proportion of different cells. Table 2 gives the percent distribution of AQP2-positive or [H^+^]-ATPase-positive or double negative cells in the cortical and medullary collecting ducts of WT and P2Y_2_ KO mice fed control or lithium-added diets for 5 months. As shown in the Figure 12 and Table 2, lithium-administration significantly decreased the percent of PC and increased the percent of IC in the WT but not in the KO mice both in the cortex and medulla (Figure 12). This in turn resulted in significant differences in the PC and IC proportions between the WT and KO mice. Accordingly, two-way ANOVA revealed significant interactions between the phenotype and lithium diet for both PC and IC cell proportions (Table 1). As shown in the Table 1, a small percent (less than 1) of cells were found to be double negative in all groups, although their proportion seem to be numerically reduced by lithium in the cortex of WT mice and medulla of KO mice. Thus, overall, it appears that genetic deletion of P2Y_2_ receptor significantly protects against lithium-induced collecting duct remodeling.

**Table 2:** Percent distribution of AQP2-positive, [H^+^]-ATPase-positive or double negative cells in the cortical and medullary collecting ducts of WT or P2Y_2_ KO mice fed control or lithium-added diets for 5 months.

Group	AQP2-positive	[H^+^]-ATPase-positive	Double negative
**Cortex**			
WT-CT	66.61 ± 1.98 (3)	32.51 ± 1.28 (3)	0.88 ± 0.88 (3)
WT-LI	55.30 ± 3.16 (4)*	44.48 ± 3.15 (4)**	0.22 ± 0.22 (4)
KO-CT	65.96 ± 4.24 (3)	33.14 ± 3.59 (3)	0.90 ± 0.90 (3)
KO-LI	69.27 ± 2.38 (4)	29.84 ± 1.79 (4)	0.89 ± 0.64 (4)
**Medulla**			
WT-CT	88.20 ± 1.80 (6)	11.43 ± 1.87 (6)	0.38 ± 0.18 (6)
WT-LI	61.80 ± 3.10 (5)^‡^	37.77 ± 3.28 (5)^‡‡^	0.46 ± 0.18 (5)
KO-CT	85.70 ± 1.80 (4)	13.73 ± 1.72 (4)	0.60 ± 0.21 (4)
KO-LI	82.20 ± 2.20 (7)	17.29 ± 2.13 (7)	0.48 ± 0.14 (7)

*Results are presented as mean ± SE of percentage values for each cell type. The numbers in the parenthesis show the number of mice examined in each group. Statistical analysis: one-way ANOVA: ^∗^significantly different from the corresponding value in the WT-CT group (*P* < 0.05) or KO-LI group (*P* < 0.05). ^∗∗^Significantly different from the corresponding value in the WT-CT group (*P* < 0.05) or KO-LI group (*P* < 0.01). ^‡^Significantly different from the corresponding value in the WT-CT group (*P* < 0.001) or KO-LI group (*P* < 0.001). ^‡‡^Significantly different from the corresponding value in the WT-CT group (*P* < 0.001) or KO-LI group (*P* < 0.001). For the results of two-way ANOVA refer to Table 1.*

### Effect of Long-Term Lithium Administration Collecting Duct Cell Proliferation

**FIGURE 13:**
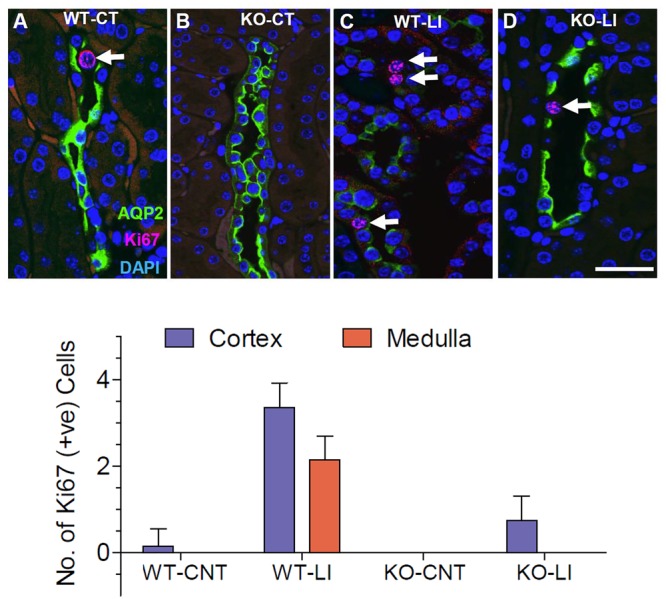
Representative profiles of immunofluorescence analysis of cell proliferation in the collecting ducts of wild type (WT) and knockout (KO) mice fed control (CT) or lithium-added (LI) diets for 5 months. **(A–D)** Representative images from the renal cortex. AQP2 immunolabeling (green) was used to identify the principal cells of the collecting duct. Sections were co-stained for the Ki67 (red) nuclear cell marker for proliferation. Nuclei are stained with DAPI (blue). Note the significantly increased number of Ki67+ cells (arrows) in lithium-treated WT mice, and the diminished AQP2 immunofluorescence in response to Li-treatment in WT, but not in KO mice. Bar is 20 μm. Bottom panel: Graphical representation of the statistical summary of the density (% of all CD cells in full frame) of Ki67+ cells in cortical and medullary collecting ducts in WT, or KO mice fed control or lithium-added diets (*n* = 4–5 each group).

Since lithium administration is known to cause collecting duct cell proliferation, we employed immunofluorescence co-labeling for AQP2 protein (green) and the nuclear antigen Ki67 (pink) in kidney sections from all groups of mice to assess the number of proliferating cells. Representative profiles of double labeling in the cortex are shown in Figure 13, where the nuclei were stained with DAPI (blue). As shown in the bar graph in Figure 13, lithium treatment caused marked increase in the number of Ki67-positive cells in both cortex and medulla of the WT mice. In the P2Y_2_ KO mice, a modest increase in the Ki67-positive cells could be seen in the cortex, but not in the medulla after lithium-treatment. Thus, it appears that genetic deletion of P2Y_2_ receptor significantly protects against lithium-induced collecting duct proliferation.

## Discussion

In this communication we documented that genetic deletion of P2Y_2_ receptor offers long-term protection against lithium-induced polyuria, natriuresis, kaliuresis, decreases in AQP2 and NKCC2 protein abundances in the renal medulla, collecting duct remodeling and proliferation of collecting duct cells. These significant findings provide a strong rationale that P2Y_2_ receptor antagonism may offer a potential treatment strategy for lithium-induced NDI (Kishore et al., 2015).

We and other investigators showed that activation of purinergic P2Y_2_ receptor antagonizes both arginine vasopressin (AVP) and aldosterone-mediated transport processes in the kidney (Rieg et al., 2007; Vallon, 2008; Wildman et al., 2008, 2009; Zhang et al., 2008, 2011; Kishore et al., 2009; Prætorius and Leipziger, 2010; Stockand et al., 2010; Vallon and Rieg, 2011). These studies established the general concept that purinergic signaling has an inhibitory effect on water and sodium absorption in the kidney. Lithium-induced NDI manifests as impaired water and sodium absorption due to resistance of the kidney to AVP action among other mechanisms. Hence, we posited that purinergic signaling may be involved in the development of lithium-induced NDI. Using a rat model of lithium-induced NDI, we first reported a link between purinergic P2Y_2_ receptor and lithium-induced polyuria (Zhang et al., 2009). Subsequently, in a 2-week model of lithium-induced NDI, we showed that genetic deletion of P2Y_2_ receptor offers significant protection against lithium-induced polyuria, natriuresis and kaliuresis, without decreasing lithium levels in the blood or in the renal medulla (Zhang et al., 2012). While these findings in the field of lithium-induced NDI were significant, it is important to note that lithium therapy in bipolar patients occurs over a long duration, years to decades. Hence, any potential new therapy for lithium-induced NDI should be examined in the context of long-term efficacy. Our model of 5-month lithium administration used here corresponds to 1/4^th^ of the average lifespan of mice or approximately equivalent to 20 years in the lifespan of humans. Assuming that there are no significant differences between genetic deletion and pharmacological blockade of P2Y_2_ receptor by a selective and potent P2Y_2_ receptor antagonist when available, extrapolation of the significant observations made here in the 5-month model support the hypothesis that P2Y_2_ receptor antagonism may be a viable option for the treatment of lithium-induced NDI in humans. In the following paragraphs we discuss the salient findings of our study and their clinical significance.

One of the salient findings is over time, lithium-induced NDI steadily became severe in the WT mice as assessed by water intake, urine output, urine osmolality, natriuresis, and kaliuresis. On the other hand, lithium-induced alterations in these parameters over time were either small or negligible in the KO mice. This finding suggests that the deleterious effects of chronic lithium administration were significantly abrogated by deletion of P2Y_2_ receptor. If this finding translates to pharmacological blockade of P2Y_2_ receptor as well, then such an approach will be very promising therapy for lithium-induced NDI.

Lithium-induced NDI is one of the few animal models wherein the AQP2 protein abundance in the renal medulla is decreased to a very low level (usually ∼20% of the control values), even after a short duration of treatment (Marples et al., 1995; Zhang et al., 2009, 2015a,b). Previously, using a 2-week model of lithium-induced NDI, we showed that KO mice had 2.4-fold higher AQP2 protein abundance in renal medulla as compared to the WT mice following lithium administration (Zhang et al., 2012). The data presented here shows that even after 5 months of lithium feeding the AQP2 protein abundance in the renal medulla of KO mice was still 2.6-fold higher as compared to the WT mice. This finding indicates the potential efficacy of suppression of P2Y_2_ receptor activity in countering the decreasing effect of lithium on AQP2 protein abundance.

Another salient feature of this long-term study is sustained suppression of lithium-induced natriuresis and kaliuresis in P2Y_2_ receptor KO mice as compared to the WT mice. Lithium-induced natriuresis is attributed to dysregulation of AVP and/or aldosterone regulated sodium channels/transporters in the kidney (Kwon et al., 2000; Grünfeld and Rossier, 2009). However, the underlying cause for lithium-induced kaliuresis is less well defined. Previously, in a 2-week model of lithium-induced NDI, we showed that medullary levels of NKCC2 and cortical levels of renal outer medullary K^+^ channel (ROMK) were not down regulated by lithium and were significantly higher in KO mice fed control or lithium-added diets (Zhang et al., 2013). Based on these findings, we suggested that increased expression of these proteins reduces Na^+^ delivery to the distal nephron and provides a buffer to attenuate collecting duct-mediated natriuresis and kaliuresis. Similar to our short-term study, here also we observed significantly higher levels of NKCC2 protein abundance in the renal medulla of lithium-fed KO mice vs. lithium-fed WT mice. The suppression of lithium-induced natriuresis and kaliuresis in the long-term model has profound clinical significance. First, it appears that suppression of lithium-induced natriuresis is unique to deletion of P2Y_2_ receptor. We reported that ADP-activated P2Y_12_ receptor is also expressed in renal collecting duct, and its pharmacological blockade by clopidogrel bisulfate or prasugrel significantly ameliorates lithium-induced NDI in rodent models. However, blockade of P2Y_12_ receptor had no effect on lithium-induced natriuresis in mice (Zhang et al., 2015a,b, 2017). Second, currently used therapeutic modalities, such as administration of amiloride or COX-2 (cyclooxygenase-2) inhibitors, do not have significant effect on lithium-induced natriuresis or kaliuresis. In fact, amiloride administration actually enhances lithium-induced natriuresis (Bedford et al., 2008; Kim et al., 2008; Kortenoeven et al., 2009). Thus, if developed into a therapeutic modality, P2Y_2_ receptor antagonism has significant and unique clinical potential to prevent sodium or potassium loss in bipolar patients on chronic lithium therapy.

At the cellular level, lithium induced NDI is characterized by collecting duct remodeling, whereby the proportion of [H^+^]-ATPase-positive or acid-secretory intercalated cells (IC) increases relative to the proportion of AQP2-positive principal cells (PC) in the kidney (Christensen et al., 2004, 2006; Welsh-Bacic et al., 2011). The cell biology of lithium-induced collecting duct remodeling is not well understood. It is know that congenital urinary tract obstruction induces changes to the renal collecting duct epithelium, including alterations and depletion of intercalated cells (Hiatt et al., 2010). Adam 10 (a disintegrin and metalloproteinase domain 10) genetic deficiency in ureteric bud also led to a reduction in the percentage of AQP2-positive principal cells (Guo et al., 2015). Inactivation of Notch signaling in the ureteric bud during embryonic development caused nephrogenic diabetes insipidus apparently by diminishing the number of principal cells and a corresponding increase in intercalated cells (Jeong et al., 2009). But to the best of our knowledge, purinergic signaling has not been shown to play a significant role in collecting duct remodeling. In this context, the findings presented here showing that genetic deletion of P2Y_2_ receptor significantly ameliorates lithium-induced collecting duct remodeling is the first piece of evidence that signaling through a G protein-coupled receptor may be involved in this process. Further in depth studies, which are beyond the scope of this communication, are needed to decipher the exact role played by P2Y_2_ receptor in lithium-induced collecting duct remodeling. Pending those studies, at this stage, our findings on the potential role of P2Y_2_ receptor in the pathophysiology of lithium-induced collecting duct remodeling further strengthen our notion that P2Y_2_ receptor antagonism is a promising and viable strategy for the treatment of lithium-induced NDI.

Lithium administration is known to cause proliferation of renal collecting duct cells (Christensen et al., 2006), which appears to be due to a decrease in cyclin-dependent kinase inhibitor p27kip1 mRNA and protein (Rojek et al., 2005). Hence, in the current study we probed for collecting duct proliferation by immunofluorescence detection of Ki67 protein, a cellular marker for proliferation. Ki67 protein is present during all active phases of cell cycle (G1, S, G2, and mitosis), but is absent from resting cells (G0) (Schlozen and Gerdes, 2000). Our results show that lithium-induced medullary collecting duct cell proliferation is significantly suppressed in the KO mice. However, further studies using appropriate methods and tools, which are beyond the scope of this communication, are needed to establish the beneficial effects of targeting P2Y_2_ receptor for the amelioration of lithium-induced collecting duct proliferation (Christensen et al., 2004, 2006).

Finally, based on the experimental data obtained *in vitro* and *in vivo*, it has been suggested that lithium treatment initiates proliferation of renal principal cells but that a significant percentage of these cells are arrested in the late G2 phase, which explains the reduced principal/intercalated cell ratio (de Groot et al., 2014). While this can be a potential possibility for the observed alterations in the PC/IC ratio in the WT mice in our study, however, it is premature to consider that lithium is not able to exert such an action in P2Y_2_ KO mice thus accounting for lack of such alterations in PC/IC ratio. It is because there is growing body of evidence showing that P2Y_2_ receptor is involved in cell proliferation (Dixon et al., 1999; Katz et al., 2011; Xie et al., 2014). Thus, further studies are warranted to decipher the exact role of blunting of P2Y_2_ receptor for the lack of lithium-induced cell proliferation vis-à-vis altered PC/IC ratio.

## Conclusion

We demonstrated that genetic deletion of P2Y_2_ receptor significantly protects mice against lithium-induced polyuria, natriuresis, kaliuresis, decreases in AQP2 and NKCC2 protein abundances, collecting duct remodeling and proliferation on long-term basis. To the best of our knowledge this is the first piece of evidence that purinergic signaling is involved in lithium-induced collecting duct remodeling and cell proliferation. We consider this communication represents a quantum leap as compared to our previous reports, where some of these features have been demonstrated in short duration (2-weeks) models.

## Ethics Statement

The animal procedures described here were approved by the Institutional Animal Care and Use Committee of the Veterans Affairs Salt Lake City Health Care System. The committee follows the guidelines of the US Public Health Service for animal experiments.

## Disclosure

The use of P2Y_2_ receptor antagonists for the treatment of lithium-induced obesity is proprietary to the United States Department of Veterans Affairs and University of Utah Research Foundation, and is protected by US Patent no. 9,901,624. Parts of this work has been presented at the Kidney Week 2014 of the American Society of Nephrology, November 2014, Philadelphia, PA, United States, and Experimental Biology 2018 meeting, April 2018, San Diego, CA, United States and appeared as printed abstract in the proceedings of that meeting (Heiney et al., 2014; Kishore et al., 2018).

## Author Contributions

YZ, JP-P, NC, and BK were involved in conception and design of research. YZ, AR-B, TL, YH, JP-P, and BK performed the experiments. YZ, JP-P, and BK analyzed the data, edited and revised the manuscript, prepared the figures, and drafted the manuscript. YZ, NC, YH, JP-P, and BK interpreted results of the experiments. YZ, AR-B, NC, TL, YH, JP-P, and BK approved final version of the manuscript.

## Conflict of Interest Statement

The authors declare that the research was conducted in the absence of any commercial or financial relationships that could be construed as a potential conflict of interest.
